# Does reflective equilibrium help us converge?

**DOI:** 10.1007/s11229-023-04375-0

**Published:** 2023-11-16

**Authors:** Andreas Freivogel

**Affiliations:** Institute of Philosophy, University of Bern, Länggassstrasse 49a, 3012 Bern, Switzerland

**Keywords:** Reflective equilibrium, Convergence, Formal modelling, Simulation study

## Abstract

I address the worry that reflective equilibrium is too weak as an account of justification because it fails to let differing views converge. I take up informal aspects of convergence and operationalise them in a formal model of reflective equilibrium. This allows for exploration by the means of computer simulation. Findings show that the formal model does not yield unique outputs, but still boosts agreement. I conclude from this that reflective equilibrium is best seen as a pluralist account of justification that cannot be accused of resulting in an “anything goes” relativism.

## Introduction

Reflective equilibrium (RE for short) is a prominent account of justification that surfaces in methodological discussions in many fields of philosophy. Although the term was coined by Rawls (1999), the idea can be traced to Goodman (1955). Very roughly, the idea of RE goes as follows: an agent starts with their initial *commitments* (“judgments”) about a subject matter. In an attempt to systematise their commitments, the agent comes up with a *theory* (“principles”) that accounts for the commitments. In a *process* of mutual adjustments, the agent revises the theory and the commitments in light of each other, striving to establish coherence among them. Typically, a *state* of reflective equilibrium is characterised in terms of coherence, e.g. by saying that commitments and theory “fit together”, which is accomplished through consistency and inferential relations.^1^ Supposedly, being held in a state of RE is what justifies the commitments as well as the theory.

Let us focus on the predominant picture of a process of equilibration leading to a state of equilibrium. This simplistic sketch of RE makes clear that RE does not start from nowhere. Inputs need to be provided to get the process of equilibration off the ground. But if agents set out from different starting points, how do the outputs of their equilibration processes relate? Do they reach the same output, or outputs that are sufficiently similar, such that we may speak of convergence?

The questions surrounding convergence in RE have emboldened critics who object to RE on the basis of a suspected lack of convergence or even the fostering of disagreement. No-convergence objections to RE are a prominent line of criticism that can be traced to the early replies to Rawls (e.g., Singer, 1974, p. 494), and the objection has been urged again and again since that time.

Most critics proceed by arguing that substantial differences in the starting points survive the process of equilibration and are preserved in the state of equilibrium because RE is overly *conservative* (Singer, 1974; Strong, 2010; Kelly & McGrath, 2010; de Maagt, 2017; Brandt, 1979; Dutilh Novaes, 2020). Other critiques do not rely on such initial differences, insisting instead that differences can arise during the equilibration process leading to divergent equilibrium states. In this regard, Bonevac (2004) and McPherson (2015) argue that equilibration processes are *path-dependent* due to the order of operations or the underdetermination of admissible adjustments.

The spectre of no-convergence is presented as a problem for the justificatory power of RE for various reasons, all of which may be summarised by the worry of Kelly and McGrath (2010) that RE is too *weak* as an account of justification. Divergent outputs reveal that RE has overly pluralistic implications. In its most extreme voicing, RE is suspected to border upon an “anything goes” relativism (de Maagt, 2017, p. 450; Haslett, 1987, p. 311). If the justificatory power of RE is staked upon its ability to produce epistemically desirable features that are commonly understood to be at odds with divergence, e.g., moral objectivity (de Maagt, 2017), then this is a serious problem. Moreover, critics fault the method of RE for not being useful in practice. Given the possibility of divergent equilibria, RE supposedly does not offer any means to resolve disagreements (Brandt, 1979, p. 22; Little, 1984, p. 383; de Maagt, 2017, p. 451).

Proponents of RE take the threat of no-convergence seriously. Extensions of the simplistic idea of RE, such as the inclusion of background theories intended to lead towards a conception of “wide RE”, can be seen as an attempt to make disagreement more “tractable” (Daniel, 1979, p. 262). Still, Tersman (2018, p. 7) finds no-convergence to be the “most troubling” objection.

Due to the highly general level at which it has tended to be discussed, descriptions of RE have remained largely as a metaphor (Hahn, 2000, 2004), and thus presented an elusive target for objections. This made it difficult to provide thorough assessments of the justificatory power of RE based on more than just metaphorical descriptions. Consequently, the treatment of convergence in RE and objections to it was left vague as well. At best, critical stances are based on plausibility considerations that draw from the formal framework of belief revision theory (Bonevac, 2004), or from the Bayesian literature (Kelly & McGrath, 2010). However, these considerations rest on general frameworks of belief change and do not stem from precise, formally worked out accounts of RE.

The aim of this paper is to address in a precise way three aspects of convergence that surface in the literature on RE. We can frame these aspects as questions: Does RE yield a unique output? Does RE promote agreement? Does RE allow for “anything” goes? In my answers, I aim to go beyond mere speculation or plausibility considerations. For this purpose, I explore the results of RE simulations on the basis of a formal model, which fleshes out the main traits of RE, and goes beyond metaphorical descriptions.

The work is organised as follows: First, I introduce the formal model and provide information about the simulations (Sect. 2). In Sects. 3, 4 and 5, I separately address the three aspects of convergence under the rubric of background-method-results-discussion. The final part (Sect. 6) serves to draw lessons for the informal debate about RE, discuss the limitations of the simulation study, and provide an outlook for further research. Some of the more technical details and robustness considerations are relegated to the appendix.

## How to simulate reflective equilibrium

### A formal model of RE

**Fig. 1:**
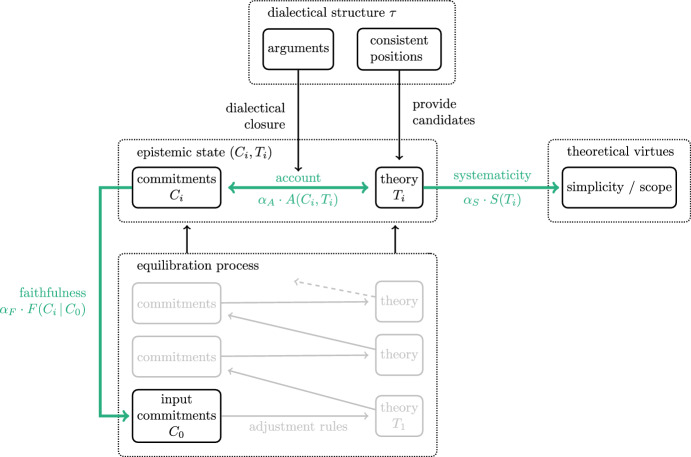
Illustrative diagram of the formal model of RE provided by Beisbart et al. (2021). The epistemic state, which consists of a set of commitments and a theory, is subject to operationalised desiderata for RE states (bold arrows). Rules for alternating adjustments of commitments and theory specify a process of equilibration that sets out from initial commitments

In order to overcome the vagueness that besets the general discussion about RE, I resort to a model provided by Beisbart et al. (2021), which represents key components of RE formally, comes equipped with a mathematically operationalised axiology of RE states, and provides rules for going through a process of mutual adjustments. Figure 1 illustrates the central components of the formal model, which draws on elaborate accounts of RE developed by e.g., Elgin (1996, 2017), and Baumberger and Brun (2017, 2021).

The epistemic state (*C*, *T*) of an agent consists of a set of commitments *C* and a theory *T*. Both components are represented as *positions* in the framework of dialectical structures (Betz, 2010, 2012), but it suffices to think of positions as sets of accepted sentences. The sentences stem from a finite pool of sentences ${\mathcal {S}}$, which is closed under negation. ${\mathcal {S}}$ is part of a dialectical structure $\tau$, which also includes a set of deductively valid arguments ${\mathcal {A}}$. We can interpret the dialectical structure to form the background of an RE inquiry. The sentence pool delineates a subject matter, and the deductively valid arguments are assumed to “comprise not just the valid arguments, but also the arguments that are valid *given the relevant background theories*” (Beisbart et al., 2021, p. 460).

A position is *minimally consistent* if and only if it does not contain flat contradictions (i.e., a sentence and its negation). On top of that, the arguments of the dialectical structure allow us to define a more demanding notion of consistency. A position is *dialectically* consistent if it is minimally consistent and satisfies all inferential relations that arise from the arguments of a dialectical structure. Sets of commitments are required to be minimally consistent, and theories must be dialectically consistent.

The following example illustrates the above definitions. The sentence pool consists of four elements, a small subset of Rechnitzer’s (2022b) reconstruction of the famous article on trolley cases by Thomson (2008).

$$s_{1}:$$
The judge must not frame an innocent person and have her executed to save five hostages from being killed by rioters.
$$s_{2}:$$
The bystander at the switch may divert the trolley so that one workman dies instead of five.
$$s_{3}:$$
The bystander on the bridge must not shove the fat man in front of the trolley in order to save the five workmen on the track.
$$s_{4}:$$
Person_A_ must let five die if saving them requires killing Person_B_.

$s_{4}$ stands in deductive inferential relations to the other sentences, forming the arguments of a dialectical structure. Inferences can be expressed as formulas: $s_{4}\rightarrow s_{1}, s_{4} \rightarrow \lnot s_{2},$ and $s_{4}\rightarrow s_{3}$. The set of initial commitments $C_{0} = \lbrace s_{1}, s_{2}, s_{3}\rbrace$ is minimally and dialectically consistent. The dialectical closure of theory $T=\lbrace s_{4}\rbrace$ is $\overline{T} = \lbrace s_{1}, \lnot s_{2}, s_{3}, s_{4}\rbrace$. However, $C_{0}$ and *T* are not dialectically consistent with each other due to the commitment $s_{2}$ and its negation as a consequence of $s_{4}$. The conflict could then be resolved in various ways, e.g., by revising the commitments or by adopting a different theory, which could lead to divergent equilibria.

Being able to resolve conflicts in different ways indicates that an agent needs to balance multiple epistemic desiderata. Desiderata are directed at the components of an epistemic state, come in degrees, and can “pull” in different directions. The bold arrows in Fig. 1 stand for three epistemic desiderata that are included in elaborate accounts of RE. At the center, we have *account*. The corresponding measure *A*(*C*, *T*) operationalises how well the commitments *C* fit to what is inferable from the theory *T* given the dialectical structure $\tau$.^2^

On the right hand side of Fig. 1, there is the desideratum of *systematicity*. A theory should “do justice to epistemic goals” in order to systematise the commitments (e.g., Baumberger & Brun, 2021, p. 7928). The operationalised measure *S*(*T*) depends on the number of elements of a theory and the number of sentences that can be inferred from it.

Finally, there is the desideratum of *faithfulness* on the left hand side in Fig. 1. It can be motivated by the view that the current commitments should “respect” the initial commitments by not changing the topic, or that a “tie” to credible or tenable initial commitments contributes to the justification of the resulting state (Beisbart et al., 2021, p. 447). $F(C \,\vert \, C_{0})$ measures the closeness of current commitments *C* and initial commitments $C_{0}$.

Trade-offs between desiderata are modelled in an achievement function that aggregates the weighted measures.

$$\begin{aligned} Z(C, T\,\vert \, C_{0}) = \alpha _{A}\cdot A(C, T) + \alpha _{S}\cdot S(T) + \alpha _{F}\cdot F(C\,\vert \, C_{0}), \end{aligned}$$

where $\alpha _{A}$, $\alpha _{S}$ and $\alpha _{F}$ are real-valued numbers between 0 and 1 that sum up to 1. The achievement function assigns to every epistemic state (*C*, *T*) a value of “overall betterness” relative to what I call an *epistemic situation* of an agent, i.e., a dialectical structure $\tau$, a set of initial commitments $C_{0}$, and a configuration of weights $(\alpha _{A}, \alpha _{S}, \alpha _{F}$).^3^ The epistemic situation captures the subject matter of inquiry, its background, and the means to handle trade-offs between epistemic desiderata. A *global optimum* relative to an epistemic situation is a state (*C*, *T*) such that there is no other epistemic state that performs strictly better according to *Z* than (*C*, *T*).

Is a global optimum according to the achievement function a state of RE? Beisbart et al. (2021, p. 449) propose additional optimality conditions on epistemic states taken from the literature on RE. For the present project, only the strongest condition is relevant:

**(FEA)**: The theory fully and exclusively accounts for the commitments.

Formally, (FEA) requires that the commitments coincide with what can be inferred from the theory given the arguments of the dialectical structure. This leads to the following definition of a full RE state:

**(Full RE state)**: An epistemic state (*C*, *T*) is a *full RE state* (relative to an epistemic situation) if and only if (i) it is a global optimum according to the achievement function *Z*, and (ii) the theory fully and exclusively accounts for the commitments (FEA).

The formal model also gives explicit rules for a process of equilibration that sets out from the initial commitments $C_{0}$. In an alternating fashion, theory and commitments are adjusted to optimise the achievement function until a stopping condition is met (for details, see Beisbart et al. (2021), p. 449). An epistemic state that results from the equilibration process is called a *fixed point*. In contrast to global optimisation, an equilibration process only considers a small fraction of all epistemic states, and hence can be seen as a “heuristic” in the sense of being a non-exhaustive search. Simulation results presented by Beisbart et al. (2021, p. 455) indicate that equilibration processes are quite successful in reaching global optima or even full RE states.

In the trolley example, an RE process starting from $C_{0} = \lbrace s_{1}, s_{2}, s_{3}\rbrace$ given the configuration of weights $(\alpha _{A}, \alpha _{S}, \alpha _{F}) = (0.55, 0.35, 0.10)$ results in a fixed point consisting of $T=\lbrace s_4\rbrace$ and $C = \lbrace s_{1}, \lnot s_{2}, s_{3}, s_{4}\rbrace$. This fixed point is a full RE state because it is globally optimal according to the achievement function and satisfies (FEA).

### The simulations

The formal model allows for implementation as a computer program.^4^ To get simulations running, *simulation setups* need to be provided, which correspond to an epistemic situation, i.e., a sentence pool and arguments that form a dialectical structure, a set of initial commitments and a configuration of weights.^5^

It is desirable to have a sample that includes many different simulation setups, but computational limitations require a trade-off between the number of dialectical structures, the number of initial commitments, and the number of weight configurations.

I will use the “two-point ensemble” to compare pairs of equilibration processes in Sects. 3 and 4. It comprises 13, 000 dialectical structures, which do not involve contentful sentences, but propositional variables and randomly generated arguments.^6^ For each dialectical structure, two random sets of initial commitments have been generated.^7^ This results in 26, 000 simulation setups.

The “full-spectrum-ensemble” is designed to investigate the allegation of “anything goes” (Sect. 5), which often takes off from the assumption that there are many and drastically different inputs. The idea of maximally diverse initial commitments can be operationalised by setting up RE simulations from the *full spectrum* of initial commitments, which consists of all non-empty and minimally consistent positions. For example, there are $3^{7} - 1 = 2{,}186$ non-empty positions that can serve as initial commitments for a sentence pool size of 7. In order to accommodate this high number of initial commitments, the second ensemble includes fewer structures (30). Table 1 presents a summary of these parameter settings and the resulting number of simulation setups.^8^

**Table 1:** The ensembles of simulations covering different variations of parameters for data generation that arise from a trade-off between the number of dialectical structures and the number of initial commitments

Ensemble name	“Two-point”	“Full-spectrum”
Weight configuration	(0.55, 0.35, 0.10)	(0.55, 0.35, 0.10)
Sentence pool size	7	7
Dialectical structures	13, 000	30
Initial commitments	2	2, 186
Simulation setups	26, 000	65, 580

The computer implementation of the formal model is able to determine fixed points, global optima and full RE states from a simulation setup. This raises the question which of the three are most relevant to the project at hand. Let me make a case for why I think that it is important to focus on *full RE fixed points*, i.e., epistemic states that are reached from a simulation setup through a process of equilibration, such that the resulting state is globally optimal according to the achievement function, and such that it satisfies (FEA).

The process of equilibration is an *imperfect* procedure, i.e., it does not guarantee that its outputs meet a process-independent “criterion of correctness” (c.f. Elgin, 1996, p. 4). This also obtains for the formal model. Some fixed points do not qualify as full RE states because they are not globally optimal, or because they do not satisfy (FEA). Arguably, justification, as a matter of yes-or-no, requires one to be in a state of full RE. Otherwise, there is room for improvement. Concerning convergence, it is most interesting to examine whether agents can reach divergent outputs that are justified. Less-than-ideal outputs cannot be used to build a case against RE if problematic features of RE outputs can be blamed on their shortcomings. However, it would be a problem if the “best” outcomes of RE, full RE fixed points in the case of the model, exhibited objectionably non-convergent behaviour.

There is also a practical upshot of considering only full RE states. Commitments are fully and exclusively accounted for by the theory (FEA). Consequently, we can look at the commitments as representative of an epistemic state. This simplifies the presentation of results considerably.

In view of my focus on full RE fixed points, I use the configuration $(\alpha _{A}, \alpha _{S}, \alpha _{F}) = (0.55, 0.35, 0.10)$, which resulted from a grid search across the parameter space. The objective of this search was to achieve full RE fixed through equilibration processes points from simulation setups (see Appendix B for details on the selection of weights and robustness considerations).

## Does reflective equilibrium yield a unique output?

### Background

Convergence to a unique output is the most straightforward entry point to explore convergence in an RE setting. Rawls (1999, p. 44) raises the question of unique outputs, but refuses to speculate about it.

In a forceful attempt to show that RE is too weak as an account of justification, Kelly and McGrath (2010, p. 337) distinguish between *intrapersonal* and *interpersonal* convergence, i.e., whether i) an individual agent with a single starting point, or ii) a group of agents with different starting points reach a unique output, respectively. Uniqueness in the intrapersonal case is a necessary but insufficient condition for interpersonal convergence.

Note that we could also subsume intrapersonal convergence as a special case under interpersonal convergence in a group of agents that share the same starting point. In this case, the question is whether agents can reach different outputs even though they set out from the same starting point.

Kelly and McGrath (2010) grant intrapersonal convergence for the sake of argument, and reject interpersonal convergence subsequently. Here, however, I would like to dwell a little more on the former; for if it turns out that intrapersonal convergence does not hold in the first place, we do not need to bother investigating interpersonal convergence on a unique output.

### Methods

Intrapersonal convergence to a unique output can be tracked easily in the formal model and its computer implementation. As it stands, the formal model does not implement interactions between agents. Epistemic states of other agents are not taken into consideration at any point in an equilibration process. Hence, we have a model of agents that engage in an intrapersonal process of equilibration.

Intrapersonal convergence might not obtain in the formal model for the following reason: Even if the model is provided with a dialectical structure, a configuration of weights and some initial commitments, some adjustments during the process of equilibration may be underdetermined. There may be multiple candidates in an adjustment step that perform equally well according to the achievement function. By design, such ties are resolved with random choices that cause an equilibration process to branch out. If we track every branch of an equilibration process, we can examine whether they lead to different fixed points.^9^

Similarly, multiple global optima can arise from ties within the achievement function. Consequently, the model might produce multiple fixed points that qualify as full RE states from a single simulation setup. In this case, the formal model would not exhibit intrapersonal convergence to a unique output.

Interpersonal convergence of two agents can be studied by considering the pairs of simulation setups in the two-point-ensemble (two sets of initial commitments in the same dialectical structure). If both simulation setups exhibit intrapersonal convergence to a unique full RE state, do they reach the same output?

### Results

**Fig. 2:**
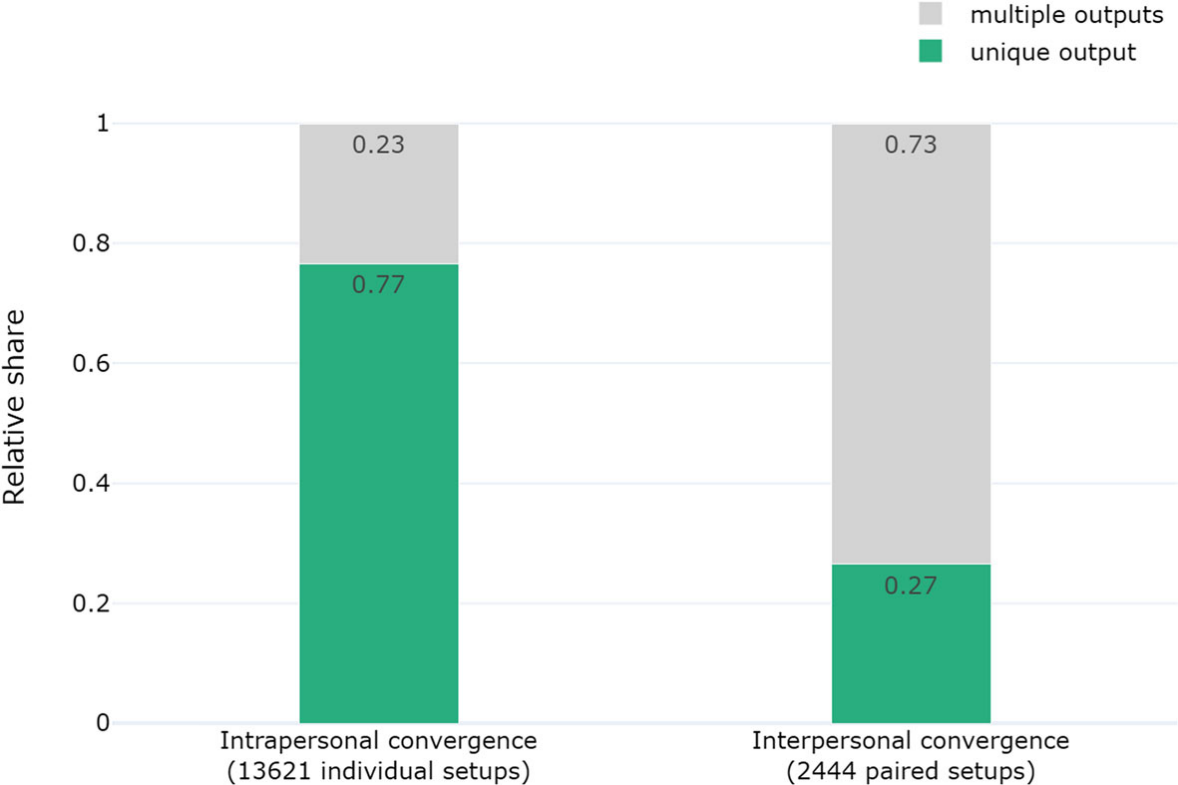
Relative shares of individual simulation setups (intrapersonal convergence) and paired setups (interpersonal convergence) that result in a unique full RE fixed point. Grey bars depict the relative share of (pairs of) simulation setups that yield more than one output

In the two-point-ensemble, 13, 621 (out of 26, 000) simulation setups yield at least one full RE fixed point. Of those, 10, 440 result in a unique output. There are 2,444 paired simulation setups in the two-point-ensemble that both yield exactly one full RE fixed point. Of those, 648 pairs reach the same output. Figure 2 depicts the relative share of (pairs of) simulation setups that yield a unique output.

### Discussion

The formal model achieves intrapersonal convergence in relatively many cases. In these cases, the formal model restricts adjustments and states so as to narrow down the range of options to a unique output. The occurrence of interpersonal convergence to a unique output from a pair of simulation setups in roughly a fourth of cases is respectable given the random generation of dialectical structures and sets of initial commitments

However, there are cases where intrapersonal and, even more so, interpersonal convergence do not obtain. Moreover, the comparison of individual to pairs of simulation setups reveals a substantive decrease in the relative share of (pairs) of simulation setups that converge to a unique output. Presumably, the model preserves some differences between the paired sets of initial commitments throughout the process of equilibration. One has to expect that considering more than two simulation setups would further erode the prospects of achieving interpersonal convergence to a unique output.

Of course, one could try to impose additional constraints in an attempt to reach unique outputs. For example, one could lower the weight for faithfulness even further or require substantial overlap in pairs of sets of initial commitments. However, I doubt whether such attempts could keep up with the intricacies of de-idealised, informal RE settings. First of all, realistic examples would contain more than seven sentences. Take for example Rechnitzer’s detailed application of RE to the justification of a precautionary principle (Rechnitzer, 2022a), which involves easily more than one hundred elements. Transferring these elements to sentences in the present formal framework would yield more than $3^{100}$ (515 septilliard) positions. Apart from not being computationally feasible, such an example bears exponentially more potential for ties, and hence might result in much more outputs. Moreover, in an informal setting, there are no ready-made numerical measures to evaluate epistemic states according to RE desiderata or straightforward solutions to handle trade-offs. Such complications might also contribute further to the multiplication of path-dependent results.

At some point, it becomes doubtful whether the constraints needed to ensure uniqueness would yield even remotely plausible constraints that would be insightful for informal applications of RE. Given the present results in a highly simplified and idealised formal model of RE, the hopes are very dim that RE in an informal setting could do better.

A more promising move is to admit that uniqueness is too stringent as a condition for convergence on RE. Uniqueness demands complete coincidence among outputs. This blocks the view of more subtle forms of agreement. As a consequence, the failure to produce a unique output gives us motivation to adopt a pluralist stance on justification with RE, as some authors already do, e.g., Elgin (1996, p. 135) or Rechnitzer (2022a, p. 236).

## Does RE promote agreement?

### Background

Instead of the uniqueness condition, we might look for a more lenient understanding of convergence in terms of “agreement” and its cognates. These notions are already in use in the literature on RE. Daniels (1979, p. 274) relates agreement to objectivity and convergence. Nielsen (1982, p. 293) describes RE as a method to achieve progress from disagreements about some initial commitments to intersubjective agreement. DePaul (2013, p. 4474) suggests that wide RE offers the means to achieve a “greater degree of agreement” among agents. Taking a critical stance towards RE, multiple outputs fail to converge if they do not fall into “a cluster of similar theories” (McPherson, 2015, p. 663), or if they are “different” (Singer, 1974, p. 494), “radically different” (Kelly, 2010, p. 339) or “conflicting” (de Maagt, 2017, p. 450).

Unfortunately, “(dis)agreement” and its cognates are highly vague notions. On many occasions, they remain undefined, and gradual and categorical readings are not distinguished from one other.

There is a notable exception, however, which offers a fruitful starting point for formalisation: Tersman (1993) distinguishes between two “systems” of beliefs being *incompatible* and *differing* from each other. According to him, two systems *A* and *B* are *incompatible* if *A* contains an element *p* such that there are elements in *B* that jointly imply that *p* is false (Tersman, 1993, p. 84). In contrast, two systems *A* and *B* *differ* if *A* contains an element that is not in *B*, or vice versa (Tersman, 1993, p. 105). As *A* and *B* may differ with respect to more or less elements, difference becomes a gradual notion.

Let us assume for the moment that we have a gradual notion of agreement at hand that is applicable to groups of inputs and outputs.^10^ We can compare initial and output agreement for a group of inputs and their resulting outputs. We may speak of convergence *to some extent* if there is more agreement among the outputs than initial agreement among the inputs.

**Fig. 3:**
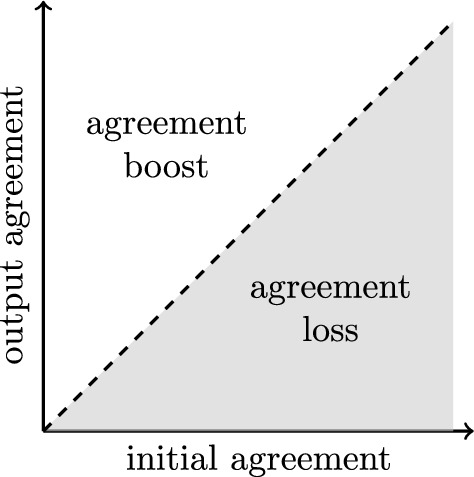
Agreement among groups of inputs (horizontal axis) and groups of outputs (vertical axis). Agreement increases in the directions of the arrows. The diagonal, dashed line indicates parity between initial and output agreement

Figure 3 displays the basic setting to spell out convergence in terms of initial and output agreement. The dashed line indicating parity between initial and output agreement separates the space into two regions. Convergence to some extent comes about if output agreement is higher than initial agreement in the upper, non-shaded area. In the lower, shaded region, output agreement does not exceed initial agreement or may even be lower than it.

Note that we could also convey convergence to unique outputs as a limiting case in this setting. If full agreement is reached if and only if the inputs converge to a unique output, then convergence to unique outputs would be a horizontal line at the very top of Fig. 3.

If RE fails to yield convergence in terms of increasing agreement, we should expect to see that RE ends up in the shaded region of the above figure in many cases.

### Methods

Tersman’s treatment of incompatibility and differences translates very well to the framework of the formal model. Compatibility amounts to the requirement that positions are consistent with each other given the arguments of the dialectical structure. Given a dialectical structure, two positions are *dialectically compatible* if and only if their set-theoretic union is dialectically consistent. In such cases, agents could aggregate their individual outputs of RE, e.g., by taking the union of their commitments, without running into contradictions. So construed, compatibility is a categorical feature of positions. It does not take the number or the severity of conflicts into consideration.

We can complement compatibility by a gradual notion of *similarity* between positions on the more fine-grained level of sentences. In the present framework, we can measure the difference between two positions on the level of sentences by a so-called *weighted Hamming distance* (see Appendix A for details). Reversing this measures operationalises similarity between positions.

The two-point-ensemble is suitable to investigate compatibility and similarity, as the operationalised measures can be applied to the paired sets of initial commitments as well as the outputs. This leads to the following setup to extract results from the data:

I restrict the two-point-ensemble to pairs of simulation setups (two sets of initial commitments in the same dialectical structure) that both yield at least one full RE fixed point. For pairs of sets of initial commitments we determine how many of them are dialectically compatible. Moreover, we calculate the similarity between positions of each pair of inputs.

As we have seen in the previous section, we need to account for the formal model producing multiple full RE fixed points per simulation setup. First, we form pairwise combinations between all full RE fixed points reached from the first and the second set of initial commitments from a pair of simulation setups.^11^ After this pairing, we determine how many of the output pairs are compatible, and calculate the similarity between the position of each pair.

### Results

There are $3{,}940 \times 2$ paired simulation setups in the two-point-ensemble that both yield at least one full RE fixed point. After pairing multiple full RE fixed point commitments from pairs of simulation setups, we arrive at 7, 368 pairs of outputs.

**Fig. 4:**
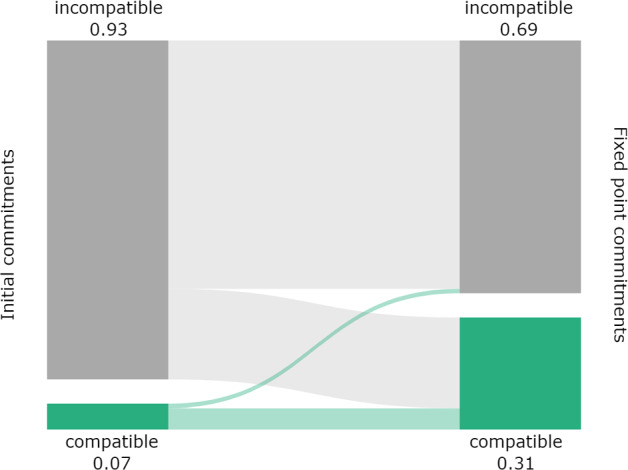
Relative share of compatible pairs of positions for initial commitments and full RE fixed points. Bands between the bars indicate the “flow” between inputs and outputs

Figure 4 gives a visual impression of the following results concerning compatibility. The relative share of compatible pairs of initial commitments is 0.07 (517 pairs), indicating that the randomly chosen initial commitments are incompatible most of the time.^12^

The relative share of pairs of full RE fixed commitments is substantially boosted to 0.31 (2, 260 pairs). Moreover, we can examine the “flow” between inputs and outputs. Most of the compatible pairs of initial commitments yield compatible pairs of outputs (relative share 0.83). Only a small portion of compatible input pairs yield incompatible pairs of outputs (relative share 0.17). A notable amount of incompatible inputs yield compatible outputs (relative share 0.27).

**Fig. 5:**
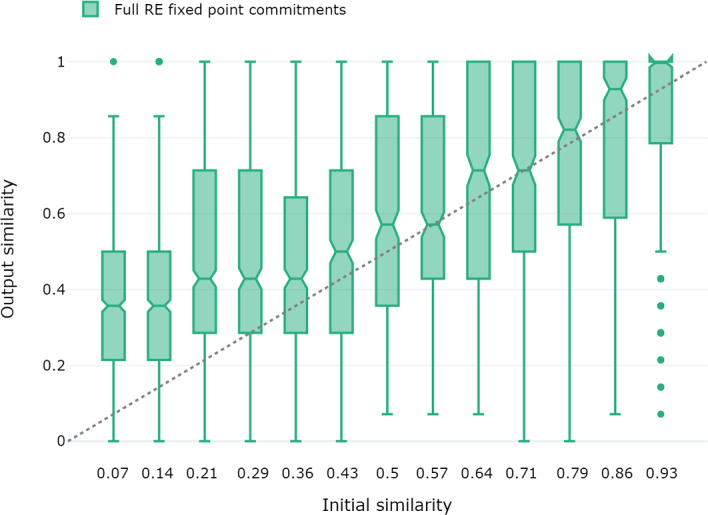
Plotting output similarity against initial similarity bins. The grey, dashed line indicates parity between input and output similarity

For similarity, the outputs have been divided into bins according to initial similarity, allowing us to plot the output similarity against these bins, as shown in Fig. 5. The boxes cover the middle 50 percent of ordered values, the interquartile range (IQR). The whiskers attached to the box have a maximal length of $1.5\cdot IQR$ (or are restricted to the most extreme actual values covered by them). Every value outside of the box and the whiskers is treated as an *outlier* represented by a dot. The horizontal line between the notches of a box indicates the median, the middle value in an ordered data set. It is more robust with respect to outliers and skew than the arithmetic mean. The notches indicate the $95\%$ confidence interval, conveying a rough visual indicator of significant differences (McGill et al., 1978).

This leads to the following observations: The median similarity among pairs of full RE fixed point commitments is slightly, yet mostly significantly higher than the similarity between the pairs of sets of initial commitments that served as inputs to produce them. Moreover, this boost of output similarity is roughly proportional to the initial similarity, but slightly more pronounced for low values of initial similarity.

### Discussion

The formal model of RE promotes agreement to some extent. Concerning compatibility, the model is able to preserve compatibility from inputs, and to establish compatibility in a substantial amount of incompatible pairs of inputs.

This result does not show that agents reach the same outputs. However, if their initial commitments are incompatible and they can aggregate their compatible fixed point commitments without running into contradictions, this can nonetheless be understood as a form of convergence. The agents reached agreement on the sentences that they both accept or reject. The remaining differences can be traced to commitments that one agent accepts or rejects, while the other agent remains silent on them.

The small relative share of compatible pairs of inputs that lead to incompatible outputs arises from the following situation. There are cases in which the only theories that account for the union of the sets of initial commitments are highly unattractive according to the measure for systematicity. Consequently, both agents choose better-performing theories that are immediately incompatible with each other. The rest of the equilibration proceeds by adjustments of commitments that “pass down” the incompatibility to the commitments before the agents settle on their respective fixed points.

Agreement, spelled out as similarity on the level of sentences, is on average slightly increased over inputs. The more agents start from similar initial commitments, the more they tend to reach similar outputs.

This is more than what the no-convergence objections would lead us to expect. In comparison to Fig. 3, if agents did not converge we would have expected that the results would tend to fall below the dashed line in Fig. 5. Now, though, we must face the question whether this is sufficient agreement. This, however, will have to be a subject for future discussion in the informal debate about RE, as critics thus far are silent on this point.

I suppose that the inclusion of systematicity into the formal model explains why the formal model is able to boost agreement. Systematicity restricts candidate theories in adjustment steps to those which strike a good balance between containing few sentences (simplicity) and entailing many (scope). The restriction of candidate theories translates to a reduced potential for incompatibility and dissimilarity. This is underwritten by additional simulations for low and high values of systematicity in Appendix B, which, for example, result in low and high relative shares of compatible outputs (Table 2), respectively. This is an important additional result. Some proponents of RE take systematisation to be the “key driver” of equilibration (Baumberger & Brun, 2021, p. 7928), but it seems to me that critics often underestimate this aspect of RE.

## Does reflective equilibrium allow for “anything goes”?

### Background

Sometimes, RE faces the charge of “anything goes”, which takes the no-convergence objection to the extreme. The worry is that the weakness of RE is so pronounced that virtually anything could be justified as (an element of) an RE output. Surveying the literature on RE reveals that we should distinguish at least between two claims about “anything goes” which are directed against RE on two different levels. On the level of sets of sentences, the objection goes that there might be as many outputs as there are inputs (de Maagt, 2017, p. 450). More precisely, the claim seems to be that the number of different sets of initial commitments is roughly equal to the number of different sets of resulting commitments. Other authors discuss “anything goes” on the level of individual sentences, which amounts to the following claim: For every belief *p* that is justified to some degree in light of cohering with a set of beliefs, there is another set of beliefs for which the negation of *p* is equally well justified (Tersman, 1993, p. 103) (see also (Elgin, 1996, p. 142)). “Anything goes” on the level of sentences is more fine-grained than on the level of sets because the former could occur even in the absence of the latter. Even if RE evaded “anything goes” on the level of sets by producing only few outputs, the outputs could still allow for “anything goes” on the level of sentences.

### Methods

The full-spectrum-ensemble is suitable to study “anything goes” as it operationalises agents that start from maximally diverse initial commitments in a dialectical structure. Again, outputs are restricted to full RE fixed points. I analyse whether “anything goes” holds in each of the 30 randomly generated structures separately, and subsequently report averages across the structures.

“Anything goes” on the level of sets can be operationalised straightforwardly as a comparison between the number of sets of initial commitments and the number of different sets of output commitments. If “anything goes” holds, we cannot expect to see a substantial reduction in numbers between inputs and outputs.

How are we to check whether “anything goes” holds on the level of sentences? We distinguish two cases according to how the inputs are grouped. First, we can take the full spectrum of initial commitments and form a single group of all outputs. For this group of outputs, we iterate through every position and keep track of every sentence that occurs. If every sentence from the sentence pool as well as its negation occur at least once, we say that the outputs *cover* the entire sentence pool, in which case “anything goes” obtains on the level of sentences. Second, we can look at initial commitments individually, and see whether there is a simulation setup that yields multiple outputs that jointly cover the entire sentence pool.

### Results

**Fig. 6:**
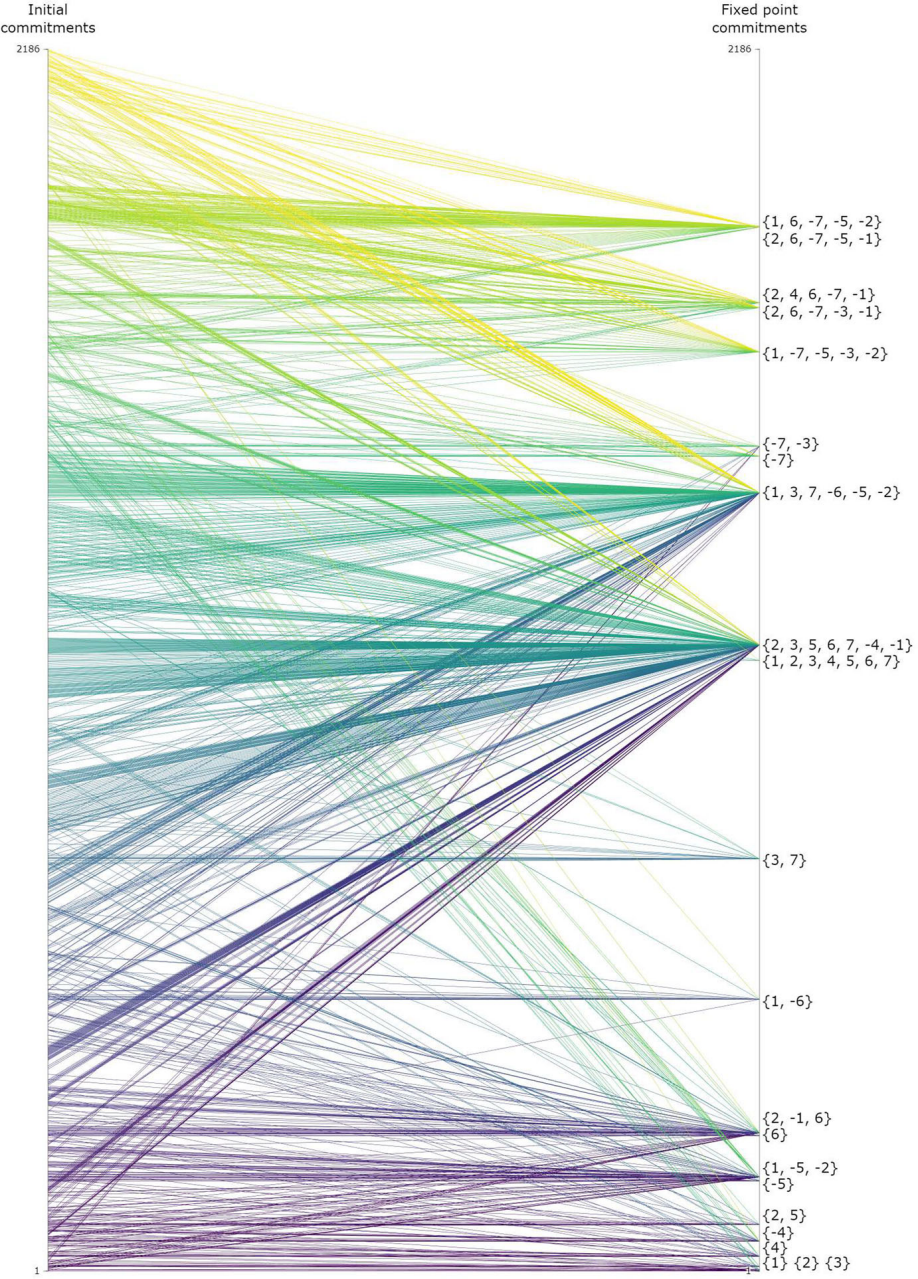
Each line connects a set of initial commitments from the full spectrum (left side) to the commitments of a full RE state reached through a process of equilibration (right side). The lines are coloured according to the sets of initial commitments ordered on a purely nominal scale. Thus, vertical height differences are, in general, not indicative of how much positions (dis)agree. Note that I use integers ($1, 2,\ldots , n$) to represent sentences instead of lower-case roman letters with indexes ($s_{1}, s_{2}, \dots , s_{n}$) for the sake of simplicity. Negated sentences ($\lnot s_{i}$) are notated as negative integers ($-i$)

On the level of sets, a drastic reduction between the number of input and output positions is apparent. This is illustrated in Fig. 6 for one dialectical structure from the full-spectrum-ensemble. From 2,186 different, minimally consistent positions that serve as initial commitments, $1,\!\!143$ reach at least one full RE fixed point through a process of equilibration in this example. There are 22 different sets of fixed point commitments that “attract” a median number of 23 sets of initial commitments (IQR: $18{-}58$).

Across all 30 dialectical structures of the full-spectrum-ensemble, the median number of different sets of initial commitments that yield a full RE fixed point is 870 (*IQR*: $600{-}1{,}024$), and the median number of unique such outputs is 22 (IQR: $20{-}24$).

Let us turn to “anything goes” on the level of sentences. First, for collecting the outputs from the full spectrum of initial commitments in a single group, the data reveals that *s* and $\lnot s$ occur as a commitment in some full RE fixed point for every sentence *s* in all dialectical structures. Take for example the outputs of Fig. 6. Each sentence as well as its negation occur in the commitments of a full RE fixed point. Thus, we have an example of a dialectical structure in which the group of outputs covers the entire sentence pool. Second, if we look at all outputs for individual sets of initial commitments, 8 of 30, 879 individual simulations produced a group of multiple outputs that covered the entire sentence pool (relative share: 0.0003).

### Discussion

The formal model drastically reduces the number of unique outputs in comparison to the number of inputs. I suppose that this dispels DeMaagt’s worry that there might be as many outputs as there are inputs. “Anything goes” does not obtain on the level of sets. Requirements and desiderata that are drawn from informal accounts of RE and implemented in the formal model thus sift out positions drastically.

On the level of sentences, there are two results that arise from grouping the outputs by guaranteeing either no or full agreement of inputs. Concerning the latter, we noticed in Sect. 3 that there is some leeway that may lead to multiple outputs from individual simulation setups. The present result establishes that, in extremely rare cases, there is enough room to allow for “anything goes” on the level of sentences when agents start from the same initial commitments.

Still, in every examined dialectical structure, sufficiently diverse sets of initial commitments reach outputs that cover the entire sentence pool, and hence allow for “anything goes” on the level of sentences.

Let me explain why I think that this result is not problematic for RE. I presume that differences in equilibria can be traced to differences in the epistemic situations of agents. In contrast to the equilibration process in the formal model, which terminates whenever the stopping condition is met, even “wider” RE does not stop there. Scanlon (2003, p. 152f) and Tersman (2018, p. 7) stress the importance of taking known disagreements among different agents into account as they may disrupt the ever-provisional equilibria. If a group of agents reaches drastically different outputs, they should be suspicious of whether they all are in a state of equilibrium, and hence evaluate their current state in view of the others. This may lead to further revisions.

The present model does not allow agents to interact with each other during equilibration or to react to reaching different outputs. This opens up a series of interesting questions for further research in formal models of RE. Which mechanisms can model such interactions or reactions? Do they lead to more consensus or polarisation among groups of agents?

## Conclusion

Exploring simulations has revealed that the formal model of RE does not behave as no-convergence objections would lead us to expect. Moreover, the results meet the expectations of proponents of RE:

[Wide reflective equilibrium] has resources that might lead inquirers toward a greater degree of agreement. Nevertheless, it seems most reasonable to expect that, in the end, [wide reflective equilibrium] will produce convergence upon a small number of alternative moral views with significant differences rather than convergence on a single view. (DePaul, 2013, p. 4474)

The formal model does not always reach intra- or interpersonal convergence to a unique output, but it promotes agreement to some extent, and the threat of “anything goes” can be kept at bay effectively.

I take this to be good news for proponents of RE. For the first time in the debate about convergence in RE, we can go beyond speculation and back up plausibility considerations by reference to computer-generated data. I cannot see a reason to think that these results are a mere artefact of formalisation, and they stem from a formal model that carefully takes up components of elaborate informal accounts of RE.

I draw the following lessons for the informal debate about RE and the possibility of providing an elaborate account of RE that is defensible against no-convergence objections in view of the present results. First, the failure to produce a unique output motivates us to adopt a pluralist stance on justification with RE. Next, the inclusion of systematicity, i.e., the consideration of theoretical virtues in RE, proved to be convergence-conducive. The demand for systematisation is more or less implicit in classic and elaborate accounts of RE, but it seems that it often escapes critics’ notice. While “systematic” or cognate terms are mentioned explicitly, these notions are often not further spelled out, and they do not play a tangible role in equilibration. Finally, simulation setups reflect the epistemic situation of an agent that engages in RE. It is a merit of RE that it forces us to be explicit about such things (Rechnitzer, 2022a, p. 241), and I propose to report equilibria relative to epistemic situations. Simulations indicate that “anything goes” does not arise from a single setup, although it does occasionally arise from collections of diverse setups. Taking differences in epistemic situations into account, however, may provoke further revisions, which keeps the threat of “anything goes” at bay.

Naturally, the present study faces limitations. It rests on examples with a very small sentence pool, for example. Unfortunately, the search space grows exponentially in the number of sentences, and thus computational feasibility is quickly exhausted. This may be mitigated by switching from the costly semi-global optimisation of the present model to locally searching variants. Such variants can handle much larger number of sentences, and they model agents that proceed in a “piecemeal” fashion, another under-explored idea that originates from Goodman (1955). However, larger sentence pool sizes prevent us from achieving global optimisation, and hence we must forfeit the determination of full RE states. Next, the formal model does not pull out all the stops. As it stands, the model does not track the tenability of initial commitments (Elgin, 1996), the independent credibility of commitments (Baumberger & Brun, 2021), or interactions between agents, which can further reduce the range of admissible inputs, outputs or adjustments, respectively.

All of this is clearly the object of further research. The present study is just a stepping stone to explore new model variants and a touch-stone for upcoming results.

## Appendix A: Technical details

The following generalises the measure of *normalised agreement* of Betz (2012, p. 39) to partial positions.^13^ I rename the measure *(normalised) similarity* to prevent confusion with my present use of “agreement”: (normalised) similarity operationalises agreement on the propositional level.

Let *n* be the size of the sentence pool, and assume that *P* and *Q* are minimally consistent positions. Then,

$$\begin{aligned} \Delta (P, Q) = \frac{D_{0,1,1,2}(P, Q)}{2n} \end{aligned}$$

is the *normalised Hamming distance* between *P* and *Q* that measures differences by summing over all sentences. It is defined as follows:

$$\begin{aligned} D_{d_0,d_1,d_2,d_3}(P, Q) = \sum _{i=1}^{n} d_{d_0,d_1,d_2,d_3}(P, Q, \lbrace s_{i}, \lnot s_{i}\rbrace ) \end{aligned}$$

where, $d_{3}, d_{2}, d_{1}$, and $d_{0}$ are penalties for contradiction, contraction, expansion, and agreement, respectively. Finally,

$$\begin{aligned} d_{d_0,d_1,d_2,d_3}(P, Q, \lbrace s_{i}, \lnot s_{i}\rbrace ) = {\left\{ \begin{array}{ll} d_{3}&{} \text {if } \lbrace s_{i}, \lnot s_{i}\rbrace \subset (P\cup Q)\\ d_{2}&{} \text {if } \lbrace s_{i}, \lnot s_{i}\rbrace \cap (P)\ne \emptyset \\ {} &{}\quad \text {and } \lbrace s_{i}, \lnot s_{i}\rbrace \cap (Q) = \emptyset \\ d_{1}&{} \text {if } \lbrace s_{i}, \lnot s_{i}\rbrace \cap (P) = \emptyset \\ {} &{}\quad \text {and } \lbrace s_{i}, \lnot s_{i}\rbrace \cap (Q) \ne \emptyset \\ d_{0}&{} \text {otherwise} \end{array}\right. } \end{aligned}$$

$1-\Delta$ is a measure of similarity. Note that the penalties of the Hamming distance $D_{0, 1, 1, 2}$ are chosen such that contradictions receive a penalty of 2, while it penalises contractions and expansions with 1. This choice reflects the idea that two positions differ to a greater extent if they contradict each other rather than if one position includes a sentence for which the other remains silent. According to this choice, the Hamming distance is normalised accordingly by the doubled size of the sentence pool 2*n*.

## Appendix B: Robustness considerations

Prospective configurations of weights $(\alpha _{A}, \alpha _{S}, \alpha _{F})$ have been determined in a grid search across the entire parameter space given the objective of reaching full RE fixed points in an independent ensemble of simulations. The results of simulations with different weight configurations are reported in Tables 2 and 3.

Overall, the results reveal the model’s robustness with respect to some variation in the weight configurations and therefore lend credibility to the findings reported in this article. There is substantial intrapersonal convergence to a unique output, an increase in compatibility and in similarity between input and output, and “anything goes” at the level of positions is blocked and occurs very rarely at the level of sentences from individual setups.

**Table 2:** Results for different weight configurations concerning the relative share of individual and paired setups that yield the same output, the relative share of compatible pairs of initial and output commitments, and the mean initial and output similarity

	Unique output		Compatibility		Similarity	
Configuration	Intrapers	Interpers	Initial	Output	Initial	Output
(0.35, 0.55, 0.10)	0.83	0.34	0.18	0.57	0.59	0.67
(0.46, 0.10, 0.44)	0.78	0.15	0.13	0.23	0.54	0.55
(0.55, 0.20, 0.25)	0.79	0.24	0.13	0.30	0.56	0.57
(0.55, 0.35, 0.10)	0.77	0.27	0.07	0.31	0.46	0.57
(0.70, 0.20, 0.10)	0.78	0.31	0.05	0.31	0.53	0.59

**Table 3:** Simulation results for additional weight configurations concerning “anything goes” on the level of positions (median number of different positions across structures), and on the level of sentences (relative share of output groups covering the entire sentence pool)

	Positions		Sentences	
Configuration	Median	IQR	Structure	Individual setups
(0.35, 0.55, 0.1)	16	16–19	1.0	0.00000
(0.46, 0.10, 0.44)	55	47–65	1.0	0.00002
(0.55, 0.20, 0.25)	33	29–35	1.0	0.00006
(0.55, 0.35, 0.10)	22	20–24	1.0	0.00026
(0.70, 0.20, 0.10)	32	27–35	1.0	0.00043

## Footnotes

1 Clearly, “equilibrium”, “coherence” or “adjustment” remain vague in this rough sketch of RE. For an elaborate account of RE that spells out six conditions on equilibrium states, see Rechnitzer (2022a, pp. 18–36) and references therein.

2 For formal details of all measures, see Beisbart et al. (2021, pp. 464–466).

3 Beisbart et al. (2021) report global optima and full RE states relative to initial commitments as the dialectical structure and the configuration of weights are held fixed. In contrast, I will vary structures and configurations as well.

4 In contrast to the original implementation in Mathematica (Beisbart et al., 2021) we developed a Python implementation, which is available at https://github.com/debatelab/tau and https://github.com/debatelab/rethon.

5 Note that other specifications of the formal model, such as the achievement function *Z*, its contributing measures, or penalties therein, are held fixed (c.f. Beisbart et al., 2021, p. 465f).

6 The random generation ensures that the resulting dialectical structure is minimally orderly. The structures have a sentence pool of size 7, and $5{-}8$ arguments consisting of $1{-}2$ premises and a conclusion. The arguments are jointly satisfiable, they are not question-begging by repeating the conclusion among premises, they avoid flat contradictions, and they do not use the same premises for different conclusions.

7 The sets of initial commitments are also randomly chosen, but it is ensured that they consist of at least three sentences, and that the number of sentences, about which positions of a pair differ, are spread out evenly instead of being normally distributed.

8 Raw data and exploratory notebooks including interactive plots are retrievable from https://doi.org/10.5281/zenodo.8402881.

9 Note that the underdetermination of adjustments is the only form of path-dependency in the formal model. An equilibration process proceeds by semi-global optimisation, i.e., the evaluation of all theory/commitments candidates in a theory/commitments adjustment step. There are no series of adjustments of individual elements in a position whose order could become relevant.

10 Note that the same could be done for the categorical notion of compatibility. This would result in four cases, one for each combination of compatible or incompatible initial commitments and outputs. No-convergence would lead us to expect that mostly incompatible outputs are reached.

11 The order does not matter as compatibility and similarity are symmetrical.

12 Recall that the formal model does not require a set of initial commitments to be dialectically consistent. The relatively high share of incompatible pairs of sets of initial commitments is due to the fact that two positions are automatically incompatible if at least one of them is dialectically inconsistent.
